# Case Report: Prompt Response to Savolitinib in a Case of Advanced Gastric Cancer With Bone Marrow Invasion and *MET* Abnormalities

**DOI:** 10.3389/fonc.2022.868654

**Published:** 2022-04-04

**Authors:** Wen Ye, Liping He, Lei Su, Zhousan Zheng, Meilin Ding, Sheng Ye

**Affiliations:** ^1^Department of Oncology, Sun Yat-Sen University First Affiliated Hospital, Guangzhou, China; ^2^Department of Geriatrics, Sun Yat-Sen University First Affiliated Hospital, Guangzhou, China

**Keywords:** savolitinib, *MET* gene, advanced gastric cancer, bone marrow invasion, case report

## Abstract

Gastric cancer is one of the most common malignant tumors and patients show a short survival, those combined with bone marrow invasion have a median survival of only 37 days. Here we reported the treatment of a 47-year-old male with advanced gastric cancer and complicated with bone marrow invasion and extensive metastases, who did not tolerate chemotherapy, under monotherapy with savolitinib, a MET receptor tyrosine kinase inhibitor. Before treatment, the patient was in severe pain and presented with thrombocytopenia and hemorrhagic anemia. Savolitinib was given based on amplification and rearrangement of the *MET* gene in his tumor. After savolitinib treatment, the patient’s condition promptly improved, efficacy evaluation indicated partial remission, and the patient was alive and remained progression-free at 15 weeks at the time of reporting. No obvious adverse reactions occurred. Besides, another case of a female gastric cancer patient with *MET* amplification who received savolitinib monotherapy as a third-line treatment that remained progression-free at 12 weeks was also reported. This report provides a new reference for understanding *MET* abnormalities in gastric cancer and offers a possibility for future application of MET tyrosine kinase inhibitors in the therapy of gastric cancer with *MET* abnormalities. Also, it suggests that sequencing of *MET* can be considered a routine target in advanced gastric cancer patients.

## Introduction

Gastric cancer (GC) is the fifth most common cancer and ranks third in cancer-related deaths (1). The median overall survival (OS) of patients with advanced GC is less than one year with combination chemotherapy (2) but if bone marrow invasion is present, the median OS is shortened to 37 days (3).

The *MET* gene-encoded protein c-mesenchymal-epithelial transition factor (MET, also known as hepatocyte growth factor (HGF) receptor) is a tyrosine kinase receptor that modulates cell proliferation, growth, survival, apoptosis, and epithelial-mesenchymal transition. Abnormal activation of MET, which promotes tumor cell proliferation and metastasis, involves *MET* exon 14 skipping mutation, *MET* amplification, and MET protein overexpression (4). Multiple studies have shown a positive relation between *MET* amplification and overexpression, and the median OS is shortened in GC patients with these abnormalities, which are considered poor prognostic factors (5–8). Savolitinib is a selective MET tyrosine kinase inhibitor (TKI) being developed for the treatment of multiple cancers. In June 2021, savolitinib was approved in China to treat progressive or intolerant local advanced or metastatic non-small cell lung cancer (NSCLC) with *MET* exon 14 skipping mutation after platinum-containing chemotherapy (9).

In this study, we reported an advanced GC patient with bone marrow invasion and extensive metastasis. Next-generation sequencing revealed that his tumor had *MET* amplification and rearrangement. After savolitinib treatment, his clinical symptoms promptly improved, no obvious adverse reactions occurred, and he was alive and remained progression-free at 15 weeks at the time of reporting. Meanwhile, another case of a female GC patient with *MET* amplification who received savolitinib monotherapy as a third-line treatment that remained progression-free at 12 weeks was also reported here. Our report demonstrated the effectiveness of savolitinib in the therapy of advanced GC with *MET* abnormalities.

## Case report

**Figure 1:**
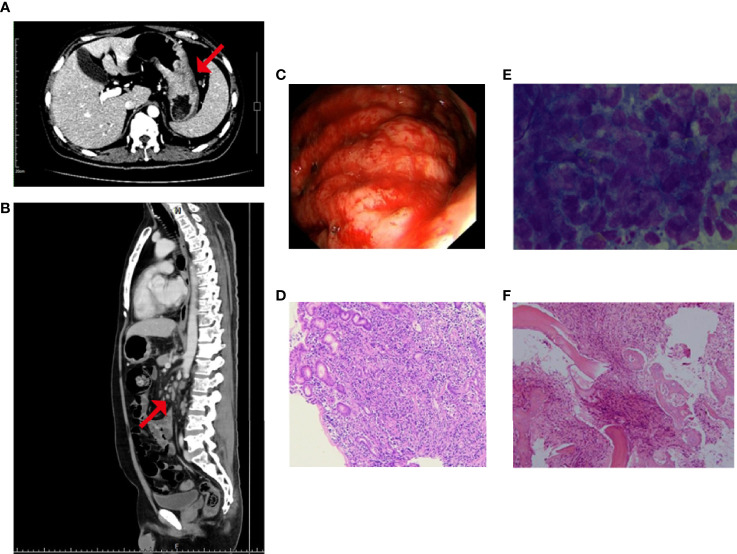
Before treatment, enhanced CT showed **(A)** thickening of gastric wall and **(B)** multiple enlarged abdominal lymph nodes, as well as mixed bone metastasis of the vertebras; the red arrow represents the location of the lesion; **(C)** Gastroscopy showed the lesion was found in the greater curvature of upper gastric body, about 5 cm × 8 cm in size, with uneven surface and bloody substances; and Hematoxylin-eosin staining of **(D)** the gastric biopsy, **(E)** the bone marrow smear, and **(F)** the bone marrow biopsy.

**Figure 2:**
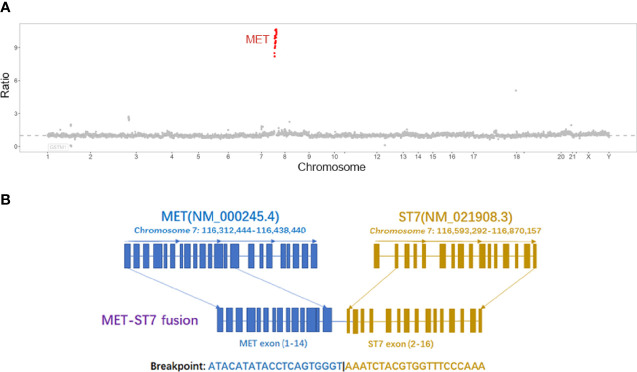
**(A)** The copy number ratio of *MET* to centromere of chromosome 7 in tissues, each red point represents an exon of *MET*; **(B)** *MET-ST7* fusion diagram.

A 47-year-old male was admitted for “low back pain and weight loss for 20 days”. He had a six-year history of type 2 diabetes mellitus and no other significant past or family medical histories. He was unable to walk at admission, with a Karnofsky performance status (KPS) score of 60 points and a pain number rating scale (NRS) score (0 to 10 points) of 8 points. Physical examination showed systemic mucosal pallor, gingival bleeding, a few ecchymoses on the right lower abdomen skin where insulin was injected, and lumbosacral vertebral tenderness. Blood test indicated severe low platelets (14 × 10^9^/L), hemoglobin (66 g/L), and normal leukocytes (6.14 × 10^9^/L). Tumor markers evaluation showed elevated CEA (103.30 µg/L), CA125 (36.00 U/mL), and CA19-9 (1321.73 U/mL). Enhanced computed tomography (CT) evidenced that the gastric fundus and body were thickened (**Figure 1A**), and multiple lymph node metastases were found in perigastric, liver hilar, retroperitoneal, mediastinal, and bilateral lung hilar regions (**Figure 1B**). Extensive mixed bone metastases throughout the body (supraorbital margin of right frontal bone, bilateral clavicles, bilateral scapulae, multiple ribs, thoracic, lumbar and sacral vertebrae, pelvic bones, right humerus, and bilateral upper femurs) were observed (**Figure 1B**). Pathological biopsy with gastroscopy (**Figure 1C**) indicated poorly differentiated adenocarcinoma (**Figure 1D**), and immunohistochemistry (IHC) of cancer cells showed HER2 negative, PD-L1 combined positive score (CPS) = 3 (Dako 22C3 antibody). Fluorescence *in-situ* hybridization showed negative Epstein Barr Virus ambiguous. A bone marrow smear (of the left posterior superior iliac spine) showed a large number of metastatic cancer cells distributed in pile, absence of megakaryocytes, and few platelets (**Figure 1E**), and a bone marrow biopsy showed cancer cells striped or scattered between bone trabeculae (**Figure 1F**). A tumor next-generation sequencing (by Geneseeq Technology Inc.) showed *MET* amplification (copies in biopsy and plasma: 19.7 and 18.1 respectively), and *MET-suppression of tumorigenicity 7* (*ST7*) rearrangement (*MET*: exon 14 - *ST7*: exon 2, in 0.8% biopsy and 5.1% plasma respectively; **Figures 2A, B** and **Supplementary Figure 1**), microsatellite stable, tumor mutation burden 6.3 mutations/Mb. Clinical diagnosis of this patient indicated stage IVB GC (complicated with bone marrow invasion, extensive bone and lymph node metastases, with *MET* amplification and rearrangement at cT4aN3M1).

**Figure 3:**
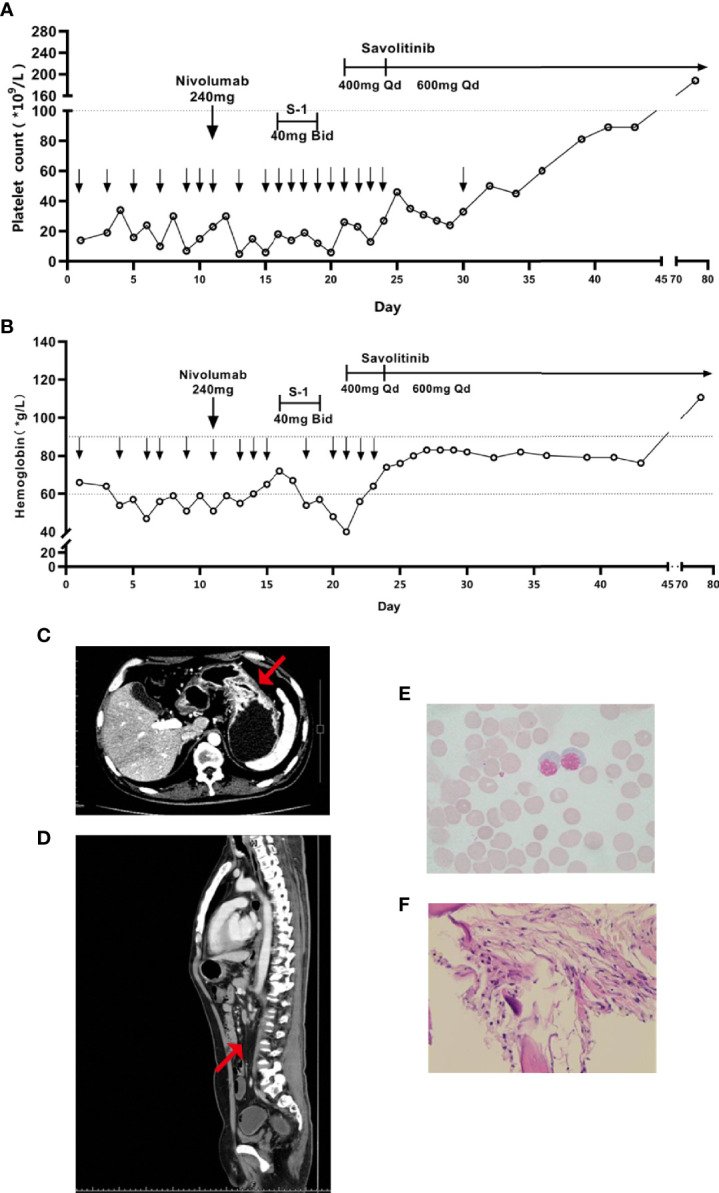
Variation trends of **(A)** platelet and **(B)** hemoglobin since admission, the lower downward arrow represents the date of platelet and hemoglobin transfusion, respectively. After savolitinib treatment, enhanced CT showed that **(C)** the thickness of gastric wall was thinner and **(D)** the abdominal lymph nodes were shrunk and fewer, the red arrow represents the location of the lesion; and post-treatment Hematoxylin-eosin staining of **(E)** the bone marrow smear and **(F)** the bone marrow biopsy.

After admission, our patient received repeated transfusions of erythrocyte, platelets, and drugs for promoting platelet production (recombinant human thrombopoietin, interleukin-11, and avatrombopag) plus supportive treatments. His hemoglobin and platelets slightly and transiently increased after blood transfusion but rapidly decreased. Eleven days after admission, the patient received one dose of 240 mg nivolumab. However, the transfusion effect declined after repeated platelet transfusion probably due to the positive anti-platelet antibodies. To block the effect of platelet antibodies, intravenous immunoglobulin (IVIG, 0.5 g/kg) was infused several times before subsequent platelet transfusion. Sixteen days after admission, induction chemotherapy with low-dose tegafur-gimeracil-oteracil potassium (*i.e.*, S-1, 40 mg, twice daily) was started when no bleeding symptoms were shown. However, three days later (nineteen days after admission), dark stools and nasal mucosal bleeding appeared, and platelet count dropped to 12 × 10^9^/L, thus S-1 was stopped. Twenty-one days after admission, the patient further developed fever, pulmonary infection, and acute left heart failure, which represented an extremely critical condition. Considering his intolerance of traditional chemotherapy as well as the significant abnormalities of the amplification and rearrangement of *MET* in his tumor, savolitinib was given to the patient (400 mg/d × 4 d, followed by 600 mg/d orally to date) along with antibiotics and supportive treatments. After savolitinib treatment for four days, platelets recovered to 27 × 10^9^/L. Bone pain symptoms were significantly relieved and the dosage of analgesics was reduced. Eighteen days after savolitinib treatment, platelets recovered to 81 × 10^9^/L, the thrombopoietic agents were stopped, then the patient was discharged. Fifty-five days after savolitinib treatment, the patient returned for a follow-up session. He was able to walk, had a KPS score of 80 points, and a pain NRS score of one point. Physical examination showed no signs of skin petechiae, ecchymoses, and lumbosacral vertebral tenderness. Blood test indicated normal platelets (188 × 10^9^/L), hemoglobin (111 g/L), and leukocytes (**Figures 3A, B****)**. Tumor markers significantly decreased (CEA 2.71 µg/L, CA125 17.2 U/mL, and CA19-9 35.04 U/mL). CT re-examination showed that gastric fundus and body wall were thinner (**Figure 3C**), and partial lymph node metastases were shrunk (**Figure 3D**). Bone marrow smear re-examination showed the absence of metastatic cancer cells (**Figure 3E**), and bone marrow biopsies showed interstitial fibrous hyperplasia with foam cell infiltration, suggesting post-treatment changes (**Figure 3F**). No obvious adverse reaction was shown. The patient achieved partial remission (PR) based on Response Evaluation Criteria in Solid Tumors (RECIST) Version 1.1. At the time of reporting, the patient was alive and remained progression-free at 15 weeks.

During the preparation of this manuscript, in our department, there was another 39-year-old female patient with advanced GC was given savolitinib. At initial admission, her pathological biopsy with gastroscopy indicated poorly differentiated adenocarcinoma, and IHC of cancer cells showed HER2 and PD-L1 CPS negative. A tumor next-generation sequencing showed *MET* amplification (9.1 copies in biopsy), microsatellite stable, and tumor mutation burden 3.95 mutations/Mb. She was diagnosed as cT4aN3M1 stage IVB GC (complicated with bilateral ovarian metastases (Krukenburg tumors), extensive lymph node metastases in perigastric, retroperitoneal, and mesenteric regions, massive abdominal-pelvic ascites, with *MET* amplification). After an 8-month first-line therapy of nivolumab plus S-1 and oxaliplatin (5-month for oxaliplatin), and a 4-month second-line chemotherapy of paclitaxel-albumin, she had a KPS score of 70 points and a pain NRS score of 4 points (abdominal pain), her primary tumor lesion and metastases markedly progressed both in size and quantity, with multiple new metastases in bones. She could not tolerate continued chemotherapy. Considering that *MET* amplification was detected in her tumor (9.1 copies), she received savolitinib monotherapy as a third-line treatment. By the eighth week, she had a KPS score of 90 points, abdominal pain relieved (NRS score of 0 points), and gained 10 kg in body weight. CA19-9 dropped from > 12000 U/ml pretreatment to 2704.83 U/ml, CEA from 26.27 ug/L to 6.73 ug/L. CT re-examination showed PR (**Supplementary Figure 2**), no obvious adverse reaction was shown. At the time of reporting, she was alive and remained progression-free at 12 weeks.

## Discussion

At present, systemic chemotherapy (including platinum and/or fluorouracil, paclitaxel, irinotecan, *etc*), immunotherapy, and targeted therapy are mainly used to treat advanced GC (10). This provided theoretical evidence for nivolumab and S-1 induction chemotherapy, which were initially selected for the male patient reported in this study. However, nivolumab efficacy was probably not achieved because of repeated infusions of IVIG.

IVIG, which contains large amounts of gamma immunoglobulin (IgG) antibodies, is often needed for the therapy of autoimmune diseases based on its contribution to anti-inflammatory and immunomodulatory activities (11). In clinical practices regarding immunotherapy-related adverse events, some patients require immunosuppressive agents including IVIG besides steroids to reduce inflammatory reactions (12–16). One of its regulation mechanisms is that IVIG can compete with pathological autoantibodies for binding the neonatal Fc receptor (FcRn). The FcRn binds serum IgG that has been endocytosed by myeloid cells or endothelial cells, and recycles the IgG to the cell surface, avoiding IgG catabolism by the lysosome (11, 17, 18). In the absence of FcRn, the half-life of IgG is reduced (19, 20). In this reported case, the transfusion effect declined after repeated platelet transfusion, which was an unfavorable condition for the patient, and we detected positive anti-platelet antibodies in the serum. We used repeated IVIG to accelerate clearance of anti-platelet antibodies. However, IVIG also could compete with nivolumab, a kind of monoclonal IgG antibody that blocks PD-1 receptors of T cells to unleash anti-tumor effect, for binding FcRn, probably resulting in the accelerated clearance of nivolumab with a shortened half-life. It is unclear at present to what extent the use of IVIG contributes to the reduction of anti-tumor efficacy of PD-1 antibodies.

Pharmacokinetics of a single dose of S-1 showed that the mean half-life of its active components are between 1.9-13.1 hours (21). Our patient received 40 mg of S-1 twice daily, which was lower than the regular dose of 60 mg according to his body surface area. Moreover, the patient suffered from thrombocytopenia, acute gastrointestinal bleeding, pulmonary infection, and heart failure. He could not tolerate continued chemotherapy after S-1 treatment for only three days.

When no standard treatments were available, the significant abnormal *MET* in the tumor of this patient attracted our attention. *MET* is located on 7q21-31. Since the 1980s, *MET* exon 14 skipping mutation, *MET* amplification, and MET protein overexpression have been associated with tumor cell proliferation and metastasis (4). In recent years, great progress has been made in research on small molecule selective MET-tyrosine kinase inhibitors (TKI), represented by savolitinib, capmatinib, and tepotinib (9, 22, 23).

Savolitinib, which showed good antitumor activities in the human glioma xenograft model, was firstly reported in 2014 as one of the inventions of a series of novel MET inhibitors (24). Being a type I small-molecule MET-TKI, savolitinib efficiently binds to the active protein kinase conformation (Asp-Phe-Gly (DFG)-in) of MET, leading to the inhibition of MET phosphorylation and downstream signaling (25). Gavine et al. reported that in a panel of GC patient-derived tumor xenograft models, savolitinib demonstrated significant anti-tumor efficacy only in MET-amplified models through the inhibition of phospho-MET and downstream signaling *via* ERK and AKT pathways (26).

To date, therapy targeting MET is mostly reported in the treatment of lung cancer, whose overall incidence of *MET* exon 14 skipping mutation as a primary driver mutation is 3% to 4%, whereas acquired *MET* amplification is detected after 5% to 20% epidermal growth factor receptor (EGFR)-TKI resistance (27). In a study conducted in 70 cases of advanced NSCLC patients with *MET* exon 14 skipping mutation, savolitinib as second-line treatment achieved an overall response rate (ORR) of 42.9%, a median progression-free survival (PFS) of 6.8 months, and a median OS of 12.5 months (28). The phase 1b TATTON study showed that combined therapy of osimertinib (an EGFR-TKI) and savolitinib, in patients with *MET* amplification secondary to *EGFR* mutation following EGFR-TKI treatment, achieved an ORR of 48% to 64% (29).

In addition to lung cancer, savolitinib is also applied for the treatment of papillary renal cell carcinoma (PRCC) and GC which is related to MET abnormalities. In a single-arm phase II study (NCT02127710) of PRCC patients treated with savolitinib, patients in the MET-driven subgroup (defined as chromosome 7 copy gain, focal *MET* or *HGF* gene amplification ≥ 6 copies, or MET kinase domain mutations > 5% allele frequency) had significant longer median PFS and higher ORR than the MET-independent subgroup (6.2 vs 1.4 months and 18% vs 0%, respectively) (30). A phase III randomized study (SAVOIR) designed solely for metastatic MET*-*driven PRCC patients favored savolitinib over sunitinib with a numerically higher response rate (27% vs 7%) (31). However, in the phase II SWOG1500 study of PRCC patients unselected for MET status, patients treated with savolitinib showed an increased risk of progression compared to those with sunitinib (32). These indicated that savolitinib exerts its highly selective antitumor activity through a MET-dependent manner.

*MET* amplification is present 1% to 10% GC (27). Previous studies revealed that *MET* amplification is involved in invasion, metastasis, advanced stage, and poor prognosis of GC. For instance, An et al. reported that the median PFS and OS of patients carrying *MET* amplification were 3.6 and 5.7 months, respectively, whereas those of patients without *MET* amplification were 6.9 and 15.5 months (33). Lee et al. found that the survival rate of GC patients with *MET* copy number greater than 4 (incidence 21.1%) was distinctly lower than that of patients with a lower copy number (8).

The VIKTORY phase II clinical trial of advanced GC confirmed that the ORR of patients with *MET* copy number greater than 10 (incidence 3.5%) treated by savolitinib monotherapy was 50%, and their PFS were 4-6 months (34). In a phase I study of capmatinib monotherapy for multiple tumors with MET overexpression or gene amplification, 2 of 9 patients with GC achieved stable disease (SD) (35). In a phase I clinical trial of tepotinib monotherapy in the treatment of multiple tumors (no limitation to *MET* status), 1 of 2 patients with GC achieved SD (36). At present, a single-arm phase II clinical trial (NCT04923932) of savolitinib for treating locally advanced or metastatic gastric cancer and esophagogastric junction adenocarcinoma with *MET* gene amplifications is ongoing.

Our male patient also had a *MET*-*ST7* rearrangement. *ST7* locates at 7q31, which is close to *MET*. *ST7* is often present with loss of heterozygosity (LOH), rather than point mutation in multiple tumors, and is believed to be a tumor suppressor gene (37–39). In a study carried out in *ALK*-rearranged positive NSCLC patients receiving ALK-TKI treatment, it was found that 3% exhibited secondary *MET-ST7* rearrangement (*MET* exons 2-21-*ST7* exon 1; the fusion site was different from that of our patient). Introducing the rearranged gene to ALK-TKI-sensitive cell line induced resistance to ALK inhibitors, and this resistance was reversed by combining treatment with MET-TKIs (crizotinib, capmatinib, or savolitinib) (40). These findings indicate that *MET-ST7* rearrangement may be pivotal in the development of cancer.

The male case reported in this article is an advanced GC patient with bone marrow invasion and extensive metastases, whose *MET* was highly amplified and co-existed with *MET-ST7* rearrangement. In the situation that routine chemotherapy was not well tolerated and the patient’s condition was extremely critical, considering the significant abnormalities of his *MET* gene as well as the published clinical data and drug accessibility, savolitinib monotherapy was selected. After savolitinib treatment, the patient’s condition promptly improved, his platelets returned to normal, anemia and bone marrow invasion disappeared. Tumor efficacy evaluation indicated PR and maintained over 15 weeks, no obvious adverse reactions occurred. We cannot completely rule out the possibility that the treatment effect is the result of a combination of all three treatments (nivolumab, S-1, and savolitinib), this is the limitation of our report. But taking into account that the patient received only one dose of nivolumab and 3-day low-dose S-1 before savolitinib treatment, accompanied by repeated IVIG which could accelerate nivolumab’s clearance, his condition was still rapidly deteriorating, we believe that the anti-tumor activity of savolitinib played a major role in his treatment strategy.

In general, this report of savolitinib monotherapy validated the prominent efficacy of savolitinib in the treatment of advanced GC with *MET* amplification, providing a potential and effective treatment option for such patients.

## Conclusion

Here we report the treatment of aggressive advanced GC patients with *MET* abnormalities, who responded to savolitinib monotherapy promptly and positively. It provides a new reference for understanding *MET* abnormalities in GC, and offers a possibility for future application of MET-TKIs in GC patients. Also, it suggests that sequencing of *MET* can be considered a routine target in advanced GC patients.

## Data Availability Statement

The original contributions presented in the study are included in the article/**Supplementary Material**. Further inquiries can be directed to the corresponding author.

## Ethics Statement

Ethical review and approval was not required for the study on human participants in accordance with the local legislation and institutional requirements. The patients/participants provided their written informed consent to participate in this study.

## Author Contributions

WY, LS, ZZ, and MD contributed to the implement of the treatment. WY and LH contributed to the collection, analysis and interpretation of data, drafting and revision of the manuscript. SY contributed to the conception of the treatment, revision and approval of the final manuscript. All authors contributed to the article and approved the submitted version.

## Funding

This work was supported by the National Natural Science Foundation of China (No.81702312) in the medical writing assistance and publication fees of the paper.

## Conflict of Interest

The authors declare that the research was conducted in the absence of any commercial or financial relationships that could be construed as a potential conflict of interest.

## Publisher’s Note

All claims expressed in this article are solely those of the authors and do not necessarily represent those of their affiliated organizations, or those of the publisher, the editors and the reviewers. Any product that may be evaluated in this article, or claim that may be made by its manufacturer, is not guaranteed or endorsed by the publisher.

## Supplementary Material

Supplementary Figure 1 Base map of *MET-ST7* fusion site.

Supplementary Figure 2 **(A)** Gastroscopy showed an ulcer lesion (black arrow) with uneven surface was found in the lesser curvature of gastric fundus, about 2.5 cm × 2 cm in size; a submucosal bulge (white arrow, proved to be poorly differentiated adenocarcinoma as well) was found at the junction of gastric fundus and body, about 1.5 cm in diameter; **(B)** Hematoxylin-eosin staining of the gastric biopsy from the ulcer lesion; Enhanced CT images of the gastric wall **(C)** at diagnosis, **(D)** progression after two lines of chemotherapy, and **(E)** after savolitinib treatment for 8 weeks. The red arrow represents the location of the lesion.
