# Supplementary figures and images for: Case Report: Prompt Response to Savolitinib in a Case of Advanced Gastric Cancer With Bone Marrow Invasion and MET Abnormalities
Source: Front Oncol. 2022 Apr 4;12:868654. doi: 10.3389/fonc.2022.868654 (PMC9013970; doi:10.3389/fonc.2022.868654)

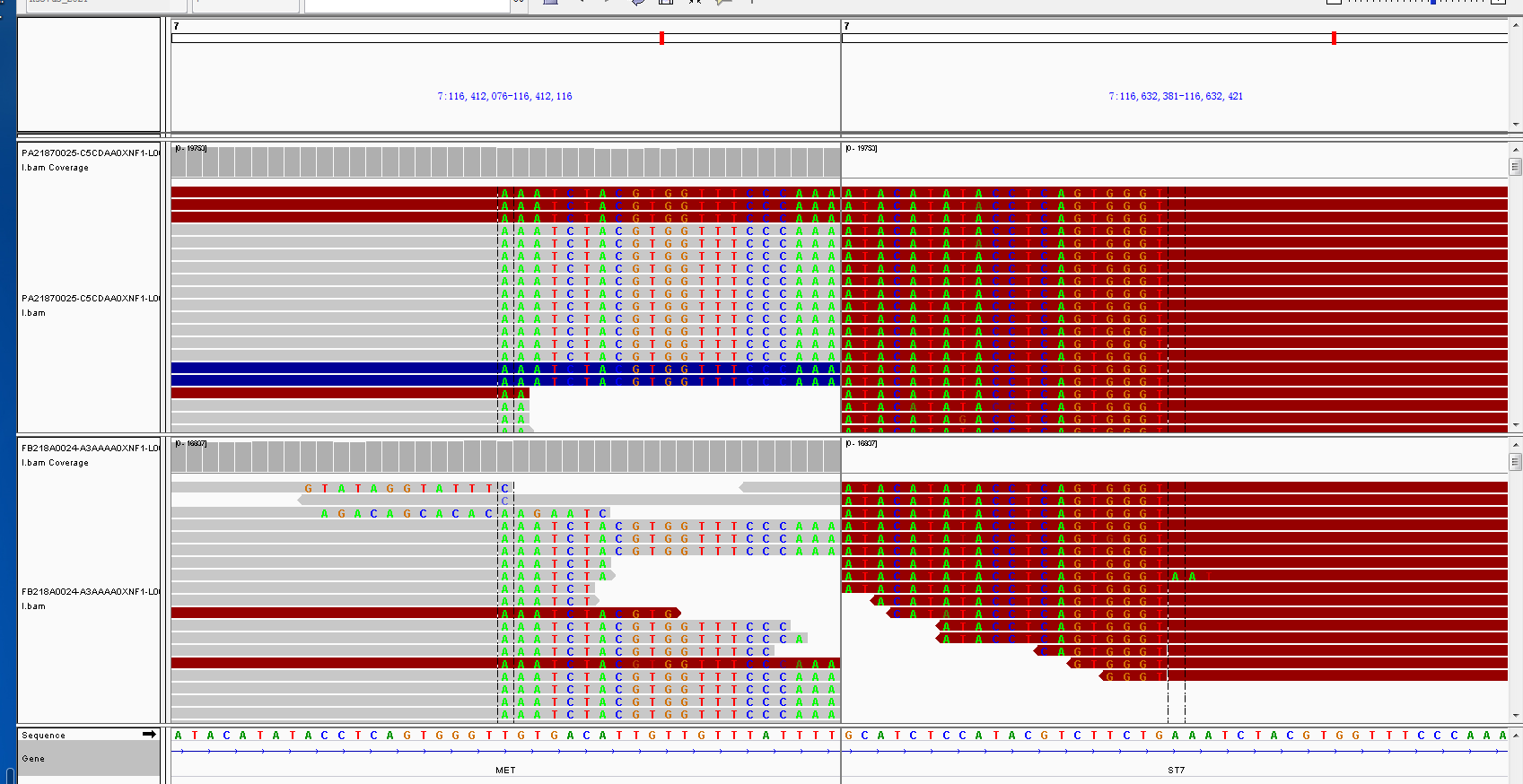

Supplement: Supplementary Figure 1 — Base map of MET-ST7 fusion site. [file Image_1.tif]

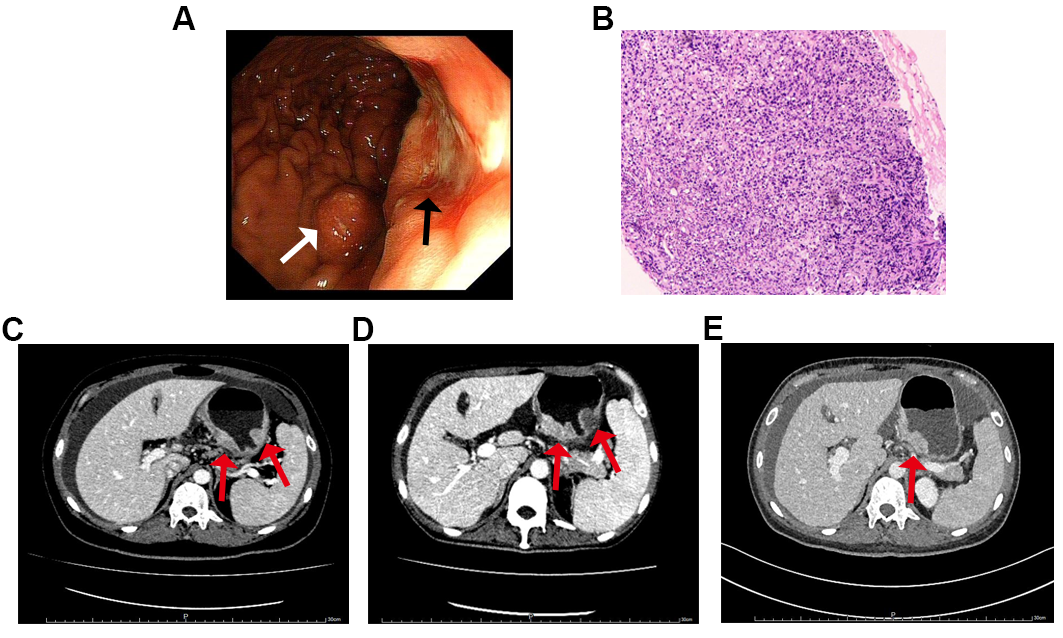

Supplement: Supplementary Figure 2 — (A) Gastroscopy showed an ulcer lesion (black arrow) with uneven surface was found in the lesser curvature of gastric fundus, about 2.5 cm × 2 cm in size; a submucosal bulge (white arrow, proved to be poorly differentiated adenocarcinoma as well) was found at the junction of gastric fundus and body, about 1.5 cm in diameter; (B) Hematoxylin-eosin staining of the gastric biopsy from the ulcer lesion; Enhanced CT images of the gastric wall (C) at diagnosis, (D) progression after two lines of chemotherapy, and (E) after savolitinib treatment for 8 weeks. The red arrow represents the location of the lesion. [file Image_2.tif]
